# Study on the Correlation Between NF-κB and Central Fatigue

**DOI:** 10.1007/s12031-021-01803-z

**Published:** 2021-02-14

**Authors:** Xingzhe Yang, Feng Li, Yan Liu, Danxi Li, Jie Li

**Affiliations:** College of Traditional Chinese Medicine, Beijing University of Chinese Medicine, Beijing, China

**Keywords:** NF-κB pathway, Central fatigue, Mechanism, Neurotransmitters, Synapse

## Abstract

In recent years, the World Health Organization (WHO) has included fatigue as a major risk factor for human life and health. The incidence rate of fatigue is high. In Europe and America, nearly 1/3 of the population is suffering from fatigue. Due to the acceleration of modern people’s life rhythm and the increase of work pressure, more and more attention has been paid to central fatigue. The activation of NF-κB is related to central fatigue, which has been paid little attention by previous studies. At the same time, previous studies have mostly focused on the immune regulation function of NF-κB, while the NF-κB pathway plays an equally important role in regulating nerve function. NF-κB can participate in the occurrence and development of central fatigue by mediating immune inflammatory response, regulating central excitability and inhibitory transmitters, regulating synaptic plasticity and regulating central nervous system (CNS) functional genes. In addition to neuroprotective effects, NF-κB also has nerve damage effects, which is also closely related to the occurrence and development of central fatigue. In this review, we focus on the relationship between NF-κB pathway and central fatigue and further explore the biological mechanism of central fatigue. At the same time, the clinical application and potential of typical NF-κB inhibitors in the treatment of fatigue were analyzed to provide reference for the clinical treatment of central fatigue.

## Background

In recent years, the World Health Organization has included fatigue as a major factor endangering human life and health. The incidence rate of fatigue is high. In Europe and America, nearly 1/3 of the population is under the threat of fatigue (Ishii et al. 2014; Otto et al. 2014). Lancet defined fatigue as the performance of obstacles in the process of carrying out or maintaining random activities (Chaudhuri et al. 2004). Fatigue is not only an independent disease, but also a symptom of many chronic diseases (Menting et al. 2018).

Central and peripheral fatigue is a common classification. Due to the acceleration of modern people’s life rhythm and the increase of work pressure, more and more attention has been paid to central fatigue. Central fatigue is caused by degenerative or other adverse changes in the central nervous system, which leads to a series of fatigue like reactions in body, nerve and psychology, including the abnormalities of emotion, cognition, learning and memory, physical function and even immune system (Han et al. 2016a). The pathophysiological process of central fatigue is related to the destruction of normal activation process of central nervous system due to metabolic disorder and structural damage (Penner et al. 2017). Abnormal regulation of immune system, damage of nerve pathway conduction function, abnormal regulation of neuroendocrine and neurotransmitter and energy loss are the core reactions of central fatigue (Leavitt et al. 2010).

The activation of nuclear factor kappa B (NF-κB) is closely related to fatigue. In addition to its important immunoregulatory function, NF-κB also plays an important role in regulating nerve function (Dresselhaus et al. 2019). The role of NF-κB in the immune system has been fully recognized in the past 20 years (Zhang et al. 2017; Razani et al. 2011; Taniguchi et al. 2018), while the understanding of the function of NF-κB in the central nervous system has just begun (Jimi et al. 2019; O’Neill et al. 1997; West et al. 2002). Studies have shown that NF-κB participates in the occurrence and development of central fatigue by regulating excitatory and inhibitory neurotransmitters, regulating synaptic information transmission, regulating synaptic plasticity and regulating central nervous system (CNS) functional genes. Due to the diversity of NF-κB complex and its activator, the diversity of target gene and the specificity of cells, NF-κB not only mediates the neuroprotective effect, but also mediates the nerve injury. Therefore, the scholars obtained the controversial results (Kaltschmidt et al. 2015). In this review, we focus on the role of activation of NF-κB pathway in the occurrence and development of central fatigue and further explore the biological mechanism of central fatigue.

NF-κB is a family of transcription factor proteins, which is the central substance of mediating immune inflammatory response (Zhang et al. 2011; Reale et al. 2018). It includes five subunits: NF-κB1 (P50), NF-κB2 (P52), RelA (P65), RelB and cRel. As a redox regulating transcription factor, nuclear factor κB mainly participates in regulating inflammation/immune response, apoptosis and cell growth (Sivandzade et al. 2019). Recent reports have demonstrated a key physiological role for the NF-ĸB signaling pathway in the central nervous system serving important functions in cellular responses to neuronal injury and synaptic plasticity (Baker et al. 2011; Hayden et al. 2012; Shih et al. 2015; Zhang et al. 2017). NF-κB can bind to IκB in an inactive state and can be activated by a variety of stimuli to regulate gene expression. NF-κB is a major transcription factor that controls the expression of many genes. It can regulate immune response (Mao et al. 2019; Mo et al. 2019) and inflammatory response (Yuan et al. 2019). By binding with ĸB (IĸB) protein inhibitor, NF-ĸB induced molecular patterns related to cytokines and pathogens and stimulated cell surface receptors, including toll-like receptors (TLRs) to trigger the activation of IB kinase complex, lead to phosphorylation, ubiquitination and degradation of IB protein. Ib proteasome degrades, resulting in release and NF-ĸB dimer is transferred to the nucleus, where it interacts with specific deoxyribonucleic acid sequence and promoting transcription of target gene eliminates the damage of operating system and cells (Sivandzade et al. 2019). NF-κB initiates the transcription of proinflammatory molecules including cytokines (IL-1, IL-6, TNF-α), cyclooxygenase-2 (COX-2), vascular adhesion molecules, inducible nitric oxide synthase (iNOS) and others (Choy et al. 2019).

## Correlation Between NF-κB Activation-Mediated Immune Inflammation Reaction and Central Fatigue

Recent studies have shown that immune inflammatory response is an important mechanism of fatigue (Manjaly et al. 2019; Dantzer et al. 2014). Cytokines released by immune cells can cause central fatigue (Suzuki et al. 2020; Conti et al. 2019).

The activation of NF-κB pathway is closely related to fatigue. Some scholars have studied the expression of microRNA in peripheral blood mononuclear cells of patients with chronic fatigue syndrome. It is found that the most significant target gene enrichment is the neuroimmune imbalance pathway (Almenar-Pérez et al. 2020) and the activation of NF-κB pathway is the key mechanism (Morris et al. 2012). Mice exposed to social stress also experienced fatigue. Compared with the control group, the immune and inflammatory pathways were enriched most significantly in the mice exposed to social stress. The up-regulated genes included tumor necrosis factor receptor superfamily gene *Tnfrsf25*, which encoded proteins that stimulated the activation of NF-κB pathway (Azzinnari et al. 2014).

Studies have shown that pro-inflammatory, inflammatory, stress-related genes and growth response are regulated by NF-κB transcription (Tilstra et al. 2012). Fatigue is associated with inflammatory markers IL6, tumor necrosis factor α (TNFα) and C-reactive protein (Bryleva et al. 2017; Akcali et al. 2017; Bower et al. 2013). NF-κB can induce transcription of cytokines TNFα, IL1β, IL6 and CRP (Utariani et al. 2020), which play an important role in the occurrence and development of fatigue. Among them, the activation of proinflammatory cytokine IL1β is the key to induce fatigue (Lampa et al. 2012). The increase of IL-1β and IL-6 may disturb the function of neurotransmitters and lead to chronic fatigue syndrome (Ma 2013). Many studies have shown that TNFα and IL1 in serum of fatigue rats are significantly increased (Han et al. 2016). It has been reported that high-intensity repeated exercise to fatigue can lead to inflammatory reaction of central nervous system, leading to the significant increase of IL-1β, IL-6, TNFα and other secretion in the brain of mice (Zhang 2015).

**Fig. 1:**
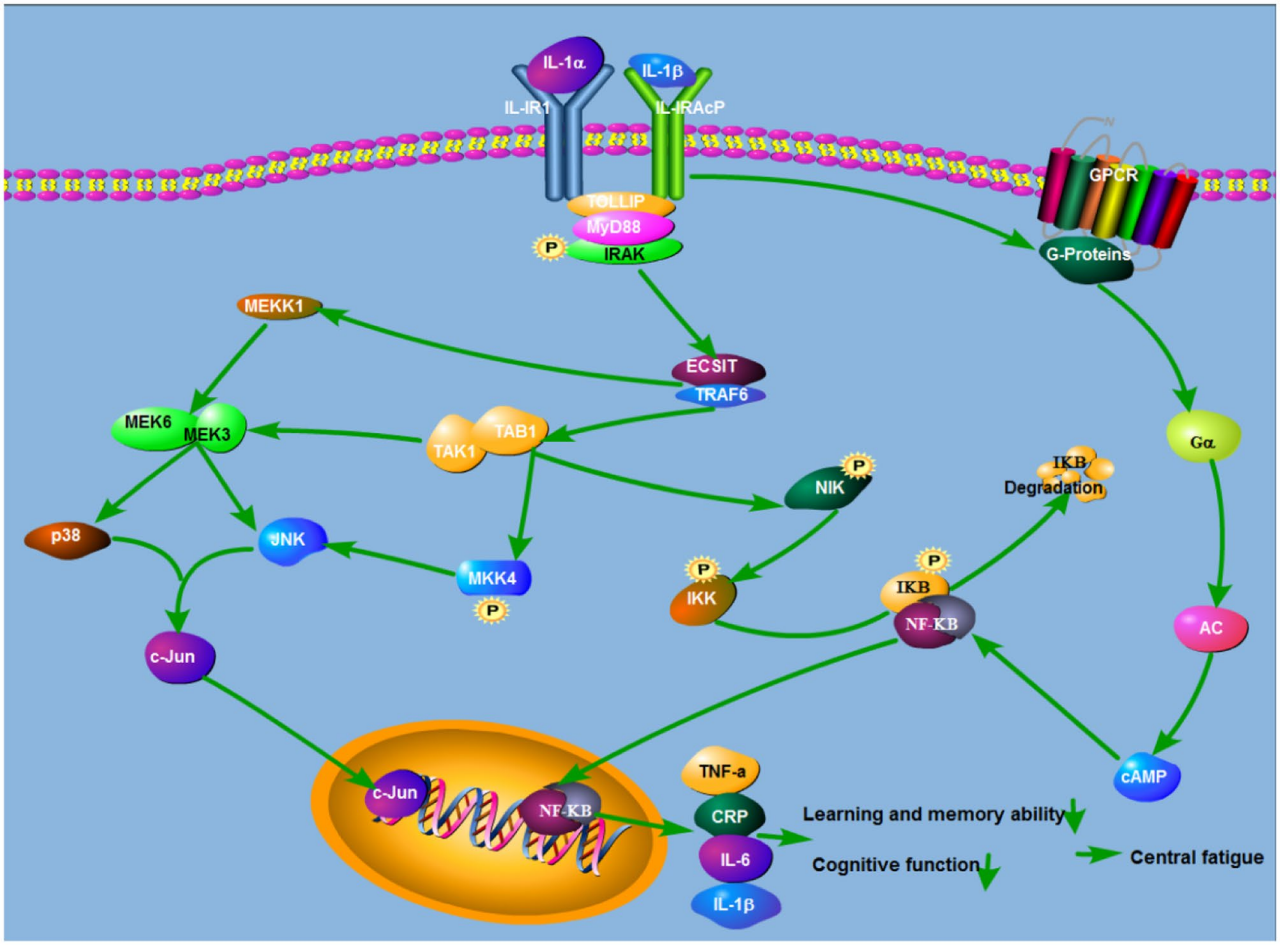
Immune inflammatory genes related to central fatigue in NF-κB pathway. Central fatigue was correlated with immune inflammatory markers IL1β, IL6, TNFα and CRP. The activation of IL1β is the key to induce fatigue. NF-κB can induce the transcription of TNFα, IL1β, IL6 and CRP, which can cause the common symptoms of central fatigue, such as the decline of learning and memory ability, cognitive dysfunction and so on

The decline of learning and memory ability is a common symptom of central fatigue. The increase of inflammatory factors is also an important mechanism leading to the decline of learning and memory ability. Many studies have shown that excessive immune activation can damage learning and memory and IL1β is the key mediator in this process (Huang et al. 2010). In addition, cognitive decline is an important consequence of fatigue. CRP is the most representative marker of nonspecific acute phase reaction caused by inflammation or tissue injury, which can induce neuroinflammatory reaction and lead to cognitive dysfunction (Lin 2010) (Fig. 1 ).

## NF-κB Participates in the Development of Central Fatigue by Regulating Excitatory and Inhibitory Neurotransmitters

Under normal circumstances, the metabolism of inhibitory and excitatory neurotransmitters in the central nervous system is in a state of balance. When the balance is destroyed, it may cause central fatigue (Zhang et al. 2006). In recent years, the imbalance of reward mechanism and inhibition of central nervous system has become a research hotspot of central fatigue mechanism, especially dopamine (DA) and gamma aminobutyric acid (GABA) that have attracted much attention as the leading substances of inhibition and reward mechanism, respectively.

The reward mechanism dominated by excitatory neurotransmitter dopamine (DA) is related to central fatigue. In the central nervous system, the process of information transmission between neurons is completed by neurotransmitters (Zhang et al. 2006). Among them, dopamine (DA) is an important excitatory neurotransmitter, which dominates the reward effect mechanism. DA can improve memory and raise working ability and efficiency, so as to relieve fatigue (McMorris et al. 2018; Ishii et al. 2014a; Tschumi et al. 2018). Ishii believed (Ishii et al. 2014a) that this reward effect mechanism can drive the brain to generate the fundamental power to work and compensate for the impact of central fatigue (Ishii et al. 2014a). Some scholars have also confirmed that the inhibition and release of DA can regulate the generation of fatigue. The decrease of DA content is an important factor causing fatigue (Han 2017). NF-κB can regulate the release of cytokines, which can affect the transmission of central nervous system. The excitatory neurotransmitter DA is very sensitive to inflammatory cytokines (Han et al. 2018) and the effect of inflammatory cytokines on ganglion dopamine is particularly related to fatigue (Azzinnari et al. 2014). NF-κB can regulate the expression of excitatory neurotransmitter dopamine by regulating the release of cytokines and affect the development of central fatigue.

**Fig. 2:**
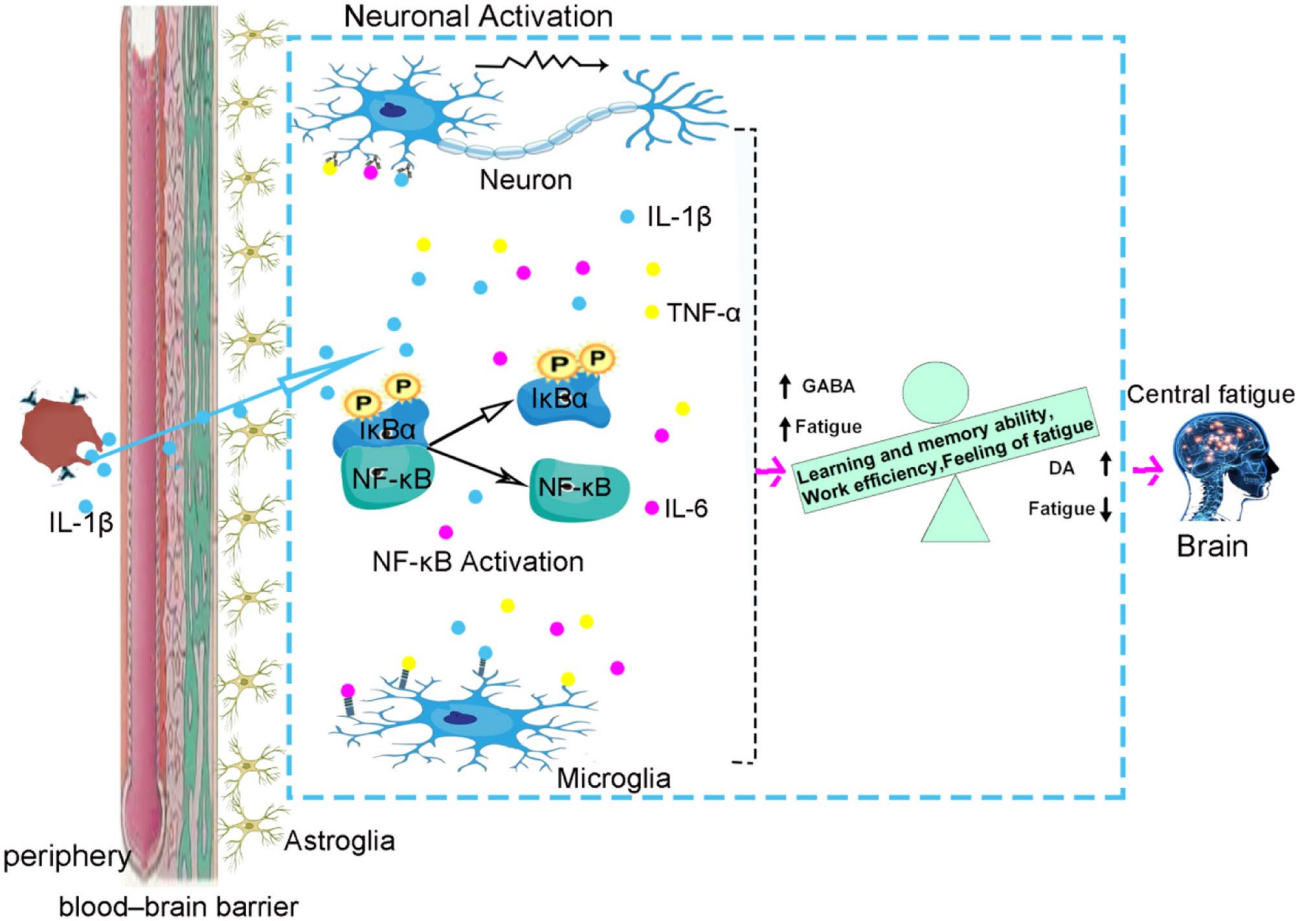
NF-κB participates in the development of central fatigue by regulating the balance between excitatory neurotransmitter dopamine (DA) and inhibitory neurotransmitter gamma aminobutyric acid (GABA). Under normal circumstances, the metabolism of inhibitory and excitatory neurotransmitters in the central nervous system is in a state of balance; when this balance is broken, central fatigue can be caused. Both the reward mechanism dominated by excitatory neurotransmitter DA and the inhibitory effect dominated by inhibitory neurotransmitter GABA are related to central fatigue. The balance between the two leading mechanisms is a hot topic in the pathogenesis of fatigue this year. NF-κB can regulate the release of inflammatory cytokines and the effect of inflammatory cytokines on dopamine in ganglion is especially related to fatigue. At the same time, NF-κB has a bidirectional regulatory effect on the neurotransmitter GABA, which affects the occurrence and development of central fatigue

The inhibitory effect of GABA is related to central fatigue. Gamma aminobutyric acid (GABA) is an important inhibitory neurotransmitter in the central nervous system (Ren et al. 2010). It has a central protective inhibitory effect to avoid brain tissue damage caused by overwork (Yang 2011). Studies have shown that the increase of GABA is an important factor leading to central fatigue (Han et al. 2016). The related experimental results also confirmed (Blanco et al. 2017) that the central nervous system of rats was in a state of inhibition after fatigue and the content of GABA was significantly increased. Glutamic acid decarboxylase (GAD65) is a rate-limiting enzyme for the synthesis of inhibitory neurotransmitter GABA. Experimental studies have shown that overexpression of NF-κB can inhibit the protective inhibitory effect of GABAergic neurons, resulting in excessive activation of excitatory neurons, which explains the reason for the enhancement of learning and memory (Kaltschmidt et al. 2015). Some studies have also confirmed that NF-κB is an important positive regulatory factor of GAD65. The expression of IκBa SR, the super inhibitory factor of NF-κB, inhibits the effect of NF-κB, resulting in the decrease of GAD65 expression and the enhancement of neuronal excitability, learning and memory. Long-term potentiation (LTP) and long-term depression (LTD) are widely regarded as the main molecular mechanisms of learning and memory. Previous studies have also shown that inhibition of NF-κB also leads to damage of LTP and LTD. In conclusion, these results suggest that NF-κB affects the occurrence and development of central fatigue through bidirectional regulation of GABA (Wierońska et al. 2010) (Fig. 2 ).

## NF-κB Regulates Synaptic Plasticity and Participates in the Development of Central Fatigue

Synapses are structures in which neurons connect with each other and transmit information. Synaptic plasticity refers to the process of adaptive changes in the structure and function of synapses under different environmental stimuli, including synaptic information transmission, synaptic development and synaptic morphological plasticity. Studies have shown that NF-κB is a key regulator of synaptic plasticity (Wierońska et al. 2010). Stimulus-coupled changes in synaptic plasticity are required for the storage, retrieval and removal of acquired information collectively referred to as memory formation. Information transmission between genes and synapses is the cellular and biochemical explanation of memory (Kandel 2001; Carmichael et al. 2018; Zhou et al. 2019). The decline of learning and memory ability and decreases of cognitive function are common manifestations of central fatigue. NF-κB plays an important role in the process of learning, memory and cognition by regulating synaptic plasticity (Snow et al. 2014; Kyrargyri et al. 2015; Vlantis et al. 2010; Sanderson et al. 2016). There are two main neurotransmitter systems in the central nervous system: glutamate (Glu) released by excitatory glutamatergic neurons and gamma aminobutyric acid (GABA) released by inhibitory glutamatergic neurons. Glutamate leads to the opening of postsynaptic Na+ channels and induces excitatory neuronal responses. On the contrary, GABA leads to the opening of Cl− channels, which induces inhibitory neuronal responses (Kaltschmidt et al. 2015). Some studies have shown that NF-κB is an important regulator of neuronal morphology. NF-κB can be activated through the basic synaptic transmission of Glu (Listwak et al. 2013; Dresselhaus et al. 2019). After activation of NF-κB, transient glutamatergic signals are transported retrogradely from the activated synapses to the nucleus and the synaptic information is transmitted to the nucleus to regulate the growth of dendrites and axons of different neuron receiving and sending structures, which is very important for long-term memory (Gorbacheva et al. 2013). NF-κB plays an important role in the regulation of synaptic information transmission and memory function.

**Fig. 3:**
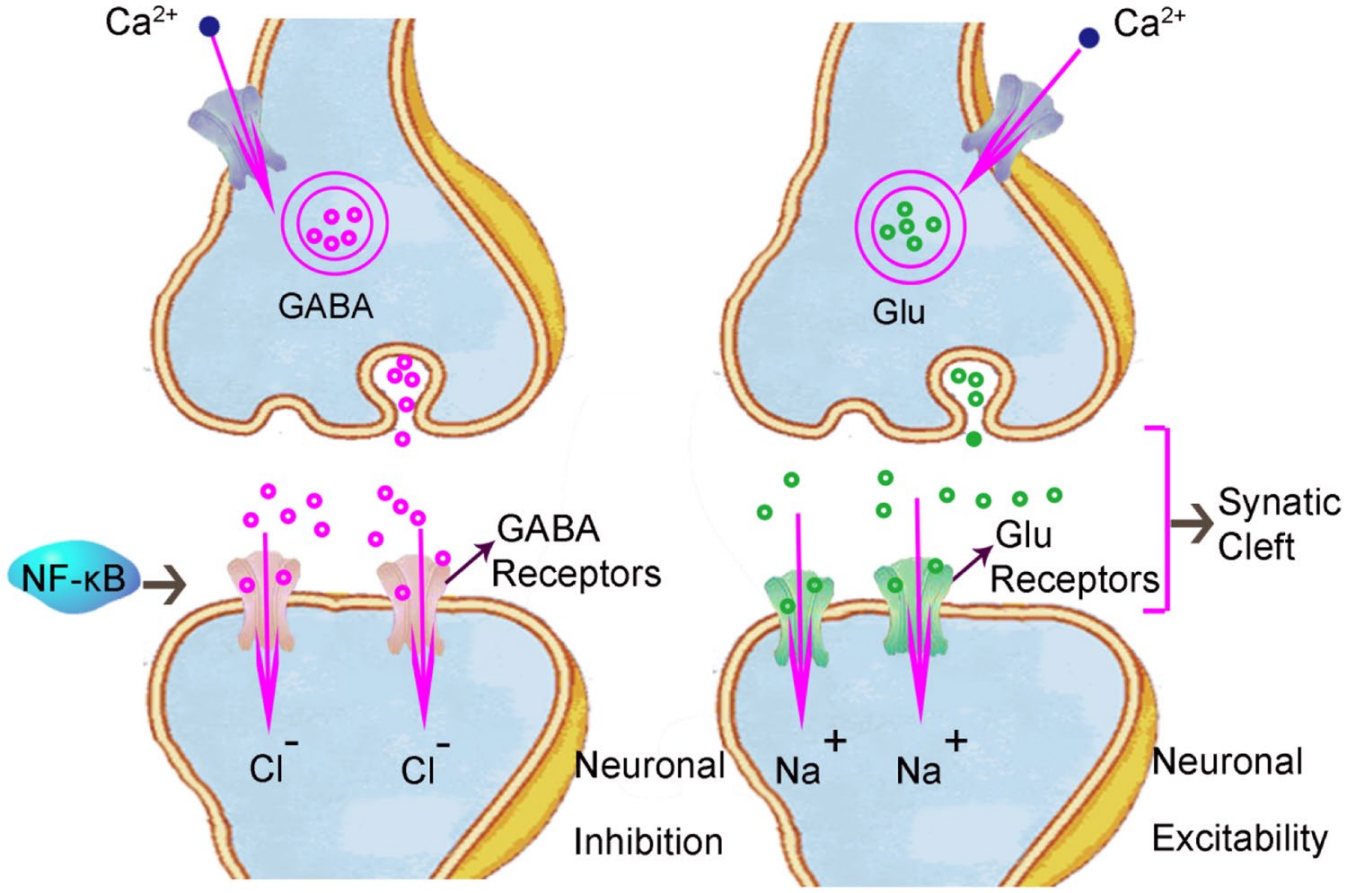
NF-κB regulates synaptic plasticity and participates in the development of central fatigue. Synaptic plasticity includes synaptic information transmission, synaptic development and synaptic morphological plasticity. There are two main neurotransmitter systems in the central nervous system: glutamate released by excitatory glutamatergic neurons and GABA released by inhibitory glutamatergic neurons. Glutamate leads to the opening of postsynaptic Na+ channels and induces excitatory neuronal responses. On the contrary, GABA induces the opening of Cl− channels and induces inhibitory neuronal responses. NF-κB is an important regulator of neuronal morphology. After being activated by the basic synaptic transmission of glutamate, NF-κB can transport transient glutamatergic signals from the activated synapses to the nucleus and transmit synaptic information to the nucleus to regulate the growth of dendrites and axons of different neurons. This is very important for the maintenance of memory ability in central fatigue

The activation of NF-κB has a positive or negative effect on the regulation of synaptic plasticity. For example, mice lacking the P50 subunit of NF-κB showed impaired learning and memory abilities and emotional disorders (Caviedes et al. 2017; Lehmann et al. 2010; Oikawa et al. 2012). On the contrary, other studies have shown that the effect of NF-κB is negatively correlated with synaptic function. For example, activation of NF-κB has also been shown to impair the production of synaptic currents in hippocampal neurons (Schwamborn et al. 2017; Kawamoto et al. 2012) (Fig. 3 ).

## NF-κB-Mediated Neuroprotective Function Is Involved in the Occurrence and Development of Central Fatigue

The pathophysiological process of central fatigue is related to the damage of nerve structure and conduction function of nerve pathway (Chaudhuri et al. 2004; Penner et al. 2017), while NF-κB has neuroprotective effect. Some scholars use the experimental study of NF-κB P50 gene-deficient mice; it has been confirmed that the activation of NF-κB has obvious neuroprotective effect on hippocampus and striatum in the prone areas of central fatigue (Oikawa et al. 2012).The neuroprotective effect of NF-κB is related to the expression of brain-derived neurotrophic factor (BDNF) (Shal et al. 2020) and erythropoietin (EPO) (Xie et al. 2012; Chong et al. 2013). In addition, activation of Janus kinase-2 (JAK2) and NF-κB resulted in transcription of NF-κB-dependent neuroprotective genes.

**Fig. 4:**
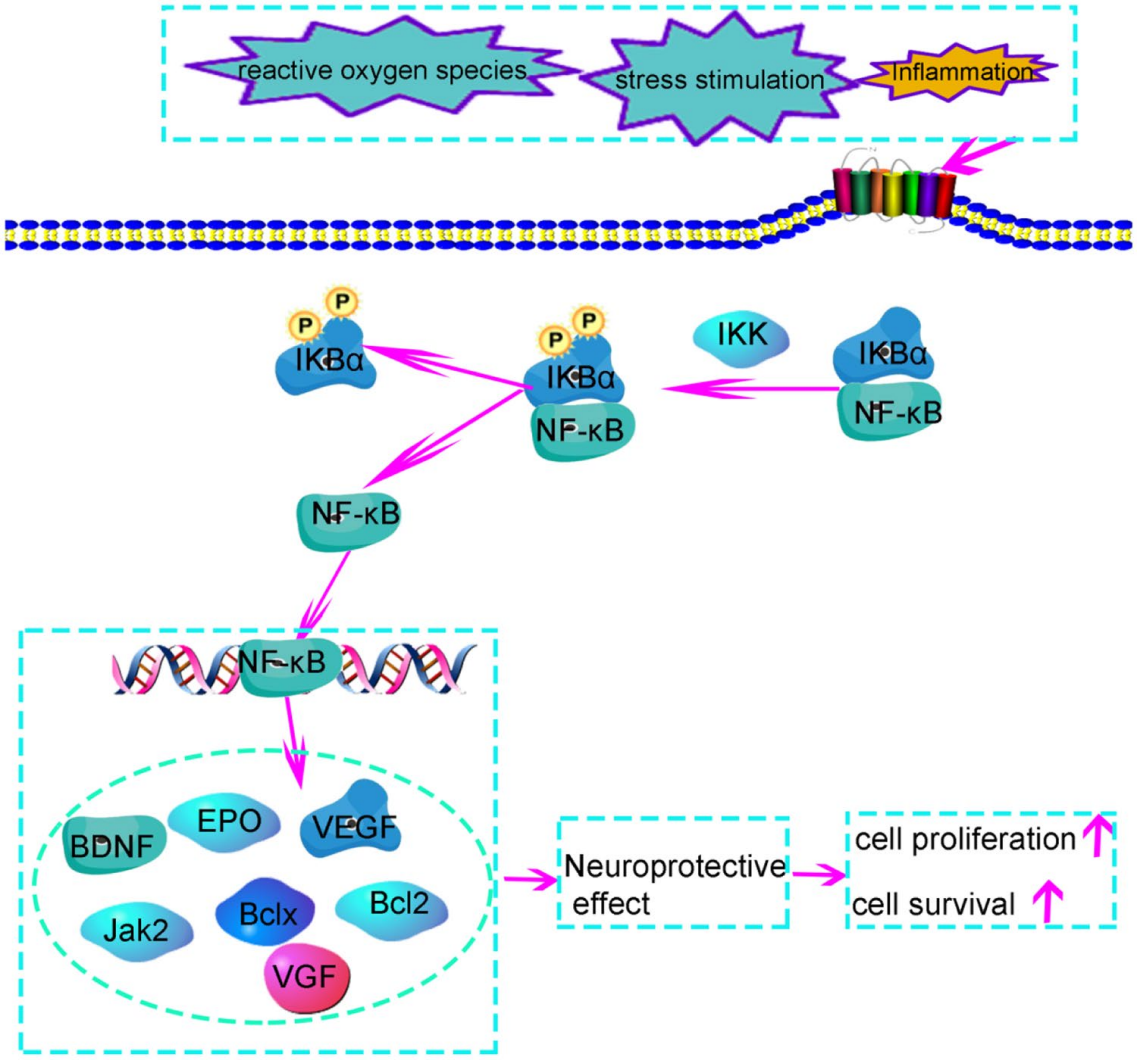
The neuroprotective effect of NF-κB is related to the pathophysiological process of central fatigue. The neuroprotective effect of NF-κB is related to the expression of brain-derived neurotrophic factor (BDNF), erythropoietin (EPO), neuroprotective genes Bcl-2 and Bcl-xl. At the same time, the activation of Janus kinase-2 (JAK2) and NF-κB led to the transcription of NF-κB-dependent neuroprotective genes. NF-κB also regulates the expression of growth factors (such as nerve growth factor (NGF), vascular endothelial growth factor (VEGF)) to promote cell proliferation and survival

NF-κB also mediates the expression of neuroprotective genes Bcl-2 and Bcl-xl and participates in the neuroprotective effect of nerve growth factor on glutamate toxicity in cultured hippocampal neurons (Casanelles et al. 2013). In addition, NF-κB also regulates growth factors (e.g., nerve growth factor, vascular endothelial growth factor) to promote cell proliferation and survival NF-κB (Zhang et al. 2017) (Fig. 4 ).

## Relationship Between NF-κB-Mediated Nerve Injury and Central Fatigue

As mentioned in previous session, central fatigue is caused by degenerative or other adverse changes in the central nervous system. Besides the neuroprotective effect, NF-κB also has nerve injury effect. The activation of NF-κB is an important mechanism of fatigue occurrence (Gallagher et al. 2014). Grilli and Memo (Grilli et al. 1999; Zhang et al. 2018) proposed that NF-κB is involved in the initiation and acceleration of various neurodegenerative processes in the process of central nervous system diseases. Many experimental and clinical studies have also proved the increase of NF-κB activity in the pathological state of central nervous system. In addition, the increase of NF-κB in astrocytes and microglia may result in neuronal degeneration through the production of ROS and proinflammatory cytokines (Morris et al. 2014).

**Fig. 5:**
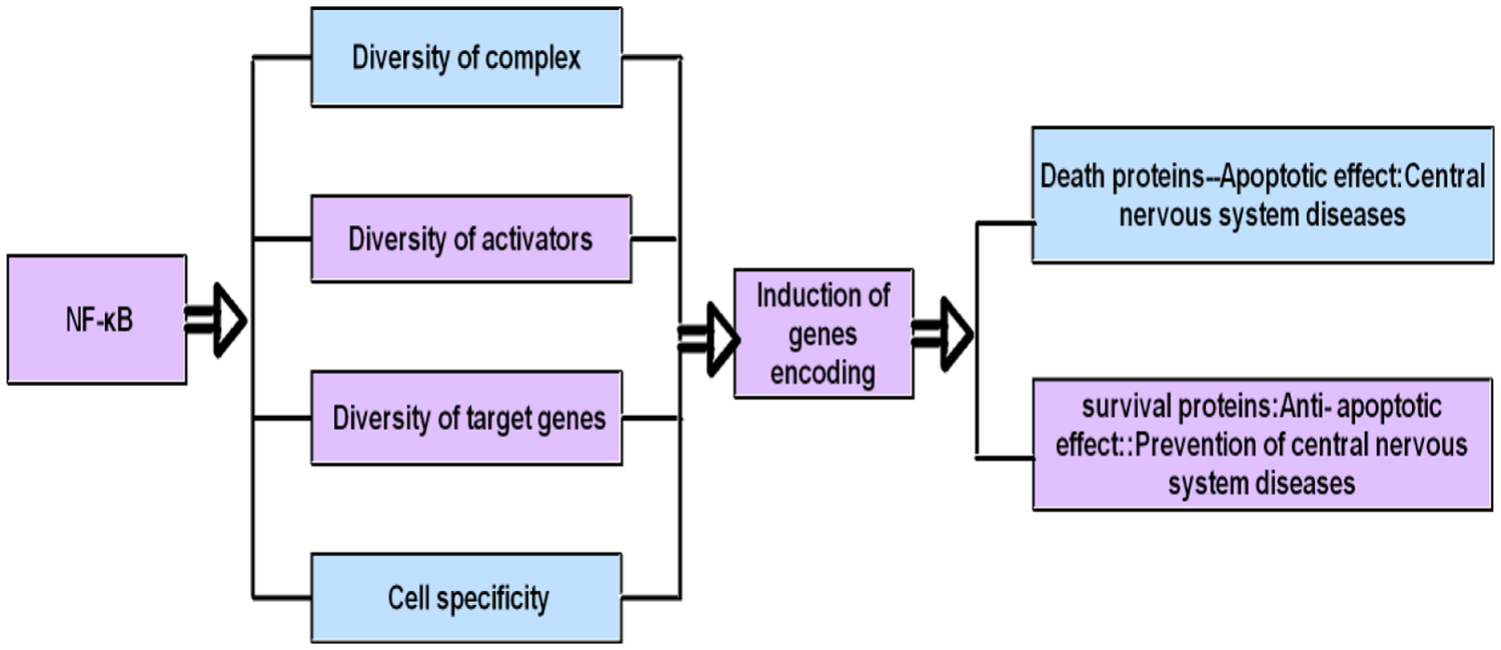
NF-κB not only has neuroprotective effect, but also has nerve injury effect. Because of the diversity of NF-κB complex and its activator, the diversity of target genes and the specificity of cells, it can induce genes encoding death or survival protein and has the effect of promoting apoptosis or anti-apoptosis. As a result, NF-κB not only has neuroprotective effect, but also has nerve injury effect and participates in the degeneration or other adverse changes of central nervous system in the process of central fatigue

These and other harmful effects may explain the important role of NF-κB production in nervous system diseases (Snow et al. 2014). According to Marwarha (Marwarha et al. 2017) and a large number of studies have shown that, due to the diversity of NF-κB complex and its activators, the diversity of target genes and the specificity of cells, under the condition of interaction with other factors, it can induce genes encoding death or survival proteins (Udovin et al. 2020), resulting in NF-κB having pro-apoptotic or anti-apoptotic effects. It is pointed out that after forebrain ischemia, the transient activation of NF-κB can cause the activation of protective factors in the surviving neurons and the continuous activation of NF-κB can induce the genes encoding death protein and lead to neuron death (Jha et al. 2019). Therefore, the use of molecules to inhibit the components of NF-κB signaling pathway is also considered as an option for the treatment of nervous system diseases, such as multiple sclerosis (Leibowitz et al. 2016), Parkinson’s disease (Wang et al. 2020), Alzheimer’s disease (Jones et al. 2017) and spontaneous intracerebral hemorrhage (King et al. 2013) (Fig. 5 ).

## NF-κB Regulates Central Nervous System Functional Genes and Affects the Occurrence and Development of Central Fatigue

NF-κB also regulates central nervous system CNS functional genes (Engelmann et al. 2014). Protein kinase A (*PKA*) and *CREB* are functional genes of central nervous system. NF-κB affects the occurrence and development of central fatigue by regulating PKA-CREB pathway. PKA is a major regulator of the nervous system, which plays an important positive role in neural development, axon growth, behavior formation and long-term memory formation (Ould et al. 2018).

Some scholars used gene chip technology to screen the related genes of exercise-induced fatigue. They found that the up-regulated genes in quadriceps femoris of exercise-induced fatigue mice included PKA (Silveira et al. 2020; Gordon et al. 2017; Caiati et al. 2013) and other protein kinase genes, which were involved in the occurrence and development of exercise-induced central fatigue (Gordon et al. 2017). CREB (protein kinase A-cAMP response element-binding protein) is a transcription factor in the nucleus, which can regulate a variety of nervous system functions, such as learning and memory. It is known as the “switch” of memory, especially in the production of growth process memory (Gruart et al. 2012; Kandel et al. 2012; Sen et al. 2019; Kim et al. 2019; Gruart et al. 2012). The activation of PKA can start the phosphorylation of CREB and then activate CREB (Park et al. 2018). Previous studies have shown that PKA-CREB pathway plays a key role in the central nervous system, which can promote the survival, regeneratio and differentiation of nerve cells and is closely related to learning and memory (Ye et al. 2017). Some studies have shown that the occurrence of fatigue is related to the increased activation of NF-κB and CREB (Black et al. 2018). Some scholars have confirmed that PKA catalytic subunit is a new target gene of NF-κB. NF-κB plays a role in learning and memory by regulating the expression of PKA in neurons and controlling CREB signaling pathway (Kaltschmidt et al. 2015; Guo et al. 2017; Gruart et al. 2012).

## Clinical Application of NF-Kb Inhibitors

NF-kB pathway plays an important role in the nervous system and the activation of NF-κB pathway is closely related to fatigue. In these circumstances, NF-kB inhibitors may be very beneficial in the treatment of central fatigue (Engelmann et al. 2014). Nrf2, an inhibitor of NF-κB, has a cerebrovascular protective effect to maintain the functional integrity of the blood-brain barrier and prevent the occurrence of neuroinflammation and degenerative CNS disorders (Li et al. 2014; Wang et al. 2014; Sivandzade et al. 2019). It can be used to treat neurodegenerative diseases and neurovascular diseases. NRF2 enhancer has been widely used in clinical practice as an inhibitor of NF-κB. For example, metformin has been also shown to concurrently inhibit NF-κB activation, thus preventing cytokine-induced expression of proinflammatory and adhesion molecule in vascular endothelial cells (Ji et al. 2016; Kim et al. 2011).

By utilizing the dual role of NRF2 activator and NF-κB inhibitor, metformin can be used in treatment regimens to reduce the burden of cerebrovascular and neuroinflammatory disease beyond the currently approved use range (type 2 diabetes) (Prasad et al. 2017; Ou et al. 2018; Tanokashira et al. 2018). Curcumin has also been shown to reduce the burden of several central nervous system diseases, including neurodegenerative diseases, global brain ischemia, intracerebral hemorrhage and TBI (Maiti et al. 2016; Alcântara et al. 2017). These beneficial effects have been linked to the activation of NRF2 (Kobayashi et al. 2016; He et al. 2013). By activating NRF2, curcumin can inhibit the activation of NF-κB and inhibit the expression of pro-inflammatory genes in microglia (Zhang et al. 2010; Li et al. 2019).

Flavonoids are biologically active polyphenolic compounds found in a wide range of fruits, vegetables, beverages (tea, coffee), nuts and cereal products with cardiovascular protective properties. According to their chemical structure, flavonoids can be divided into flavonoids, flavonoids, flavonols, flavono-3-alcohols, isoflavones and anthocyanins. Flavonoids are NF-κB inhibitors that regulate the expression of pro-inflammatory genes, thereby reducing inflammatory responses in various cardiovascular pathologies (Choy et al. 2019).

Fatigue has been associated with increased inflammatory activation of the immune system affecting both the periphery and the central nervous system (CNS) (Lee et al. 2019). The inflammatory immune response and cytokine levels have been associated with fatigue in a large body of literature across different disorders (Bower et al. 2014; Felger et al. 2013; Fung et al. 2013; Dowell et al. 2016; Eyre et al. 2016; Green et al. 2017). Based on this, we can speculate that metformin and curcumin, common activators of NRF2, may have the potential to treat fatigue. Flavonoids in the treatment of cardiovascular and cerebrovascular diseases at the same time may also have a therapeutic effect on fatigue.

## Conclusion

NF-κB is an important immune and inflammatory pathway. Previous studies have focused on the immunoregulatory function of NF-κB, while NF-κB pathway plays an equally important role in regulating neural function. The pathogenesis of central fatigue is related to the activation of NF-κB signaling pathway, which has been paid less attention in the past. NF-κB participates in the occurrence and development of central fatigue by regulating excitatory and inhibitory neurotransmitters, synaptic plasticity and functional genes of central nervous system. At the same time, NF-κB has neuroprotective and nerve injury effect, which is involved in the occurrence and development of central fatigue. It is concluded that NF-κB participates in the occurrence and development of central fatigue and has neuroprotective and nerve injury effects, which is involved in the occurrence and development of central fatigue. So, NF-κB might be a therapeutic target for the treatment of fatigue.

It may be very beneficial to use NF-kB inhibitors to treat fatigue. Dimethanidine can suppress NF-κB activation, which prevents the expression of vascular inflammation and adhesive molecules induced by cell factors. Through the activation of NRF2, turmeric has the effect of preventing NF-κB from activating and suppressing the expression of inflammatory genes in small glue cells. As one of the NF-κB inhibitors, acetone compound can regulate the expression of inflammatory genes, thereby reducing the inflammatory response under various cardiovascular pathologies. Dimethanidine, turmericin and carbone compounds have the potential to treat fatigue.
